# HOSPICE CLINICIANS’ WILLINGNESS TO BE PRESENT DURING MEDICAL AID IN DYING: A CONTENT ANALYSIS OF RATIONALES

**DOI:** 10.1093/geroni/igad104.0416

**Published:** 2023-12-21

**Authors:** Todd Becker, John Cagle, Cindy Cain, Nancy Kusmaul, Joan Davitt, Paul Sacco

**Affiliations:** University of Maryland, Baltimore, Baltimore, Maryland, United States; University of Maryland, Baltimore, Baltimore, Maryland, United States; University of Alabama at Birmingham, Birmingham, Alabama, United States; University of Maryland Baltimore County, Baltimore, Maryland, United States; University of Maryland, Baltimore, Baltimore, Maryland, United States; University of Maryland, Baltimore, Baltimore, Maryland, United States

## Abstract

Although clinician presence during medical aid in dying (MAID) may facilitate relief for patients, federal, state, and organizational policy discourage presence. Given the lack of direct examination, we aimed to describe hospice clinicians’ willingness to be present during MAID and categorize their rationales. This cross-sectional analysis used Qualtrics data collected from a convenience sample of 75 hospice clinicians. We recruited participants through hospice and palliative care membership associations for physicians, nurses, social workers, and chaplains. A quantitative item asked if participants would be willing to be present with a patient during MAID, given certain safeguards (yes, no, unsure). Participants explained their rationales in an open-ended probe. Erlingsson and Brysiewicz’s (2017) four-step content analysis was applied to open-ended rationales grouped under each previously tabulated quantitative response. The categories that we constructed for participants willing to be present (73%) reflected a commitment to patient-centered care, desire to attenuate potential medical complications, and professional responsibility despite personal discomfort. We interpreted responses from those unwilling to be present (16%) through categories of wholesale moral objections to MAID, moral objections specifically to one’s participation, and perceived incongruence between MAID and hospice care. We categorized the responses of participants who were unsure (11%) as either competing pressures or fears of personal and professional recourse. Although most participants were willing to be present during MAID, open-ended rationales revealed great nuance within each overarching quantitative response. Participants’ reported tensions with themselves, their profession, and society reflect a need for greater professional guidance and support as MAID legalization expands.

